# Diffuse Large B-cell Lymphoma of the Ovaries Presenting With a Paraneoplastic Sclerotic Skin Lesion

**DOI:** 10.7759/cureus.54974

**Published:** 2024-02-26

**Authors:** Nicholas J Garza, Kavitha Arulmozhi, Christopher LePhong, Ruetima Titapiwatanakun, Karen S Fernandez

**Affiliations:** 1 Pediatrics, Valley Children’s Healthcare, Madera, USA; 2 Pathology and Laboratory Medicine, Valley Children’s Healthcare, Madera, USA; 3 Pediatric Oncology, Valley Children’s Healthcare, Madera, USA

**Keywords:** dlbcl, scleroderma, rare presentations, paraneoplastic syndromes, ovarian lymphoma, ovarian tumor

## Abstract

Paraneoplastic presentations are often the initial presenting symptom of a malignant process. A 15-year-old female presented with a progressively growing, sclerotic lesion of the neck restricting the range of motion. She was found to have bilateral ovarian tumors that proved to be diffuse large B-cell lymphoma (DLBCL). After starting cyclophosphamide, vincristine, and prednisone (COP), she had a rapid and complete resolution of the sclerotic lesion, as well as a favorable response to the neoplastic process. In this report, we present a very rare case of extranodal lymphoma associated with a paraneoplastic skin lesion.

## Introduction

Pediatric non-Hodgkin lymphoma (NHL) accounts for 8% of childhood malignancies with diffuse large B-cell lymphoma (DLBCL) comprising 10%-20% of all NHL [1-3]. Extranodal NHL is rare and most frequently found in the bone. Primary ovarian lymphoma (POL) accounts for 0.5% of all NHL and 1.5% of all ovarian tumors [4]. The most common pediatric ovarian NHL reported in the literature is Burkitt lymphoma [5]. Paraneoplastic syndromes have been reported with lymphomas and ovarian tumors, ranging from nephrotic syndrome to encephalitis, among others. In the case of lymphomas, most of the paraneoplastic processes are associated with immune dysregulation and are antibody-mediated. Some of the dermatologic paraneoplastic syndromes described in the literature include pemphigus ichthyosis, vasculitis, and pyoderma gangrenosum scleroderma. Here, we present an unusual case of bilateral POL presenting with a paraneoplastic skin lesion that resolved with chemotherapy treatment.

## Case presentation

A previously healthy 15-year-old female presented with a progressive neck lesion for 2-3 months. It started as a 2 cm erythematous lesion on the right neck and worsened over four months, becoming more indurated and tender and extending down to the midsternal region. The induration significantly restricted the neck’s range of motion. The patient also reported mild dysphagia and six months of amenorrhea.

**Figure 1:**
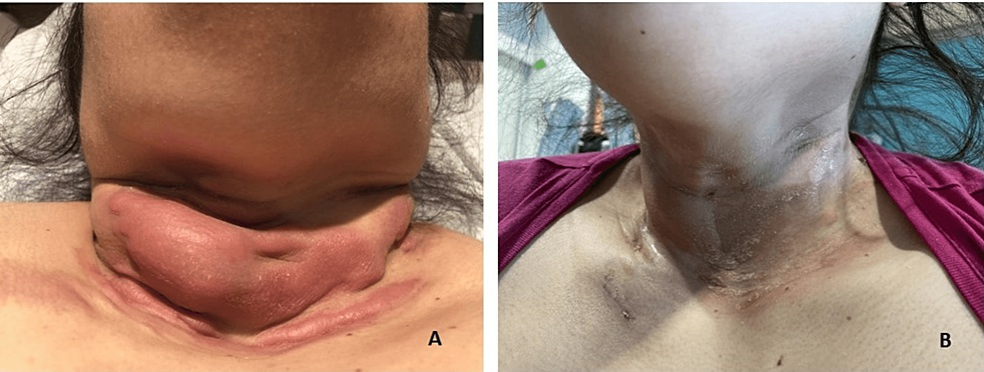
Clinical presentation of paraneoplastic skin lesion (A) Sclerotic lesion at the time of diagnosis. (B) Near resolution of the lesion after seven days of COP reduction COP: cyclophosphamide, vincristine, and prednisone

On examination, the patient was obese with a large keloid-like lesion in the lower neck (Figure 1A). The skin around the posterior neck, shoulders, and proximal chest was hard and indurated. Palpation of lymph nodes and the spleen was difficult due to body habitus. Laboratory evaluation showed a normal blood count, lactate dehydrogenase of 454 U/L, uric acid of 6.8 mg/dL, and mild transaminitis.

**Figure 2:**
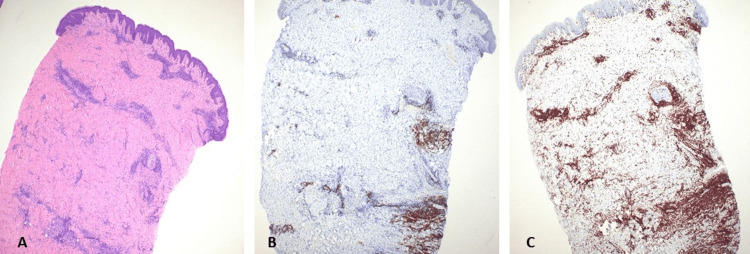
Right neck skin punch biopsy (A) H&E stain: 4×; low-power view demonstrating lymphoid aggregates surrounding the vessels, salivary glands, and hair follicles. (B) H&E stain: 4×; CD3 immunohistochemical stain highlighting a predominance of T-cells. (C) H&E stain: 4×; CD20 immunohistochemical stain highlighting scattered aggregates of B-cells H&E, hematoxylin and eosin; CD, cluster of differentiation

A computerized tomography (CT) scan of the head and neck showed a non-specific infiltrative process within the soft tissues of the neck. Full-thickness skin biopsy showed perivascular, periadnexal, and dermal-subcutis junctional lymphocytic inflammatory infiltrates. The lymphocytes consisted mostly of mature reactive T-cells with cluster of differentiation (CD) 4+ and CD8+ subsets in roughly equal proportions (Figure 2). There was significant dermal fibrosis, which in conjunction with the distribution of lymphocytes was concerning for possible early scleroderma/morphea. An extensive infectious disease and rheumatology workup was negative. CT of the chest, abdomen, and pelvis was performed to assess for an occult malignant process and uncovered bilateral ovarian masses with retroperitoneal and mediastinal adenopathy. Serum tumor markers including alpha-fetoprotein (AFP), beta-human chorionic gonadotropin (b-HCG), inhibin, and carcinoembryonic antigen were normal. However, cancer antigen 125 (CA125) was elevated at 103 U/mL (upper limit of normal {ULN}: 38.1 U/mL).

Considering lymphoma in the differential diagnosis, a laparoscopic incisional ovarian biopsy was performed. Unfortunately, the tumor cells showed severe crush artifact precluding definitive histologic identification, but the possible expression of CD34, terminal deoxynucleotidyl transferase (TdT), CD10, and paired-box gene 5 (PAX5) favored an immature B-cell neoplasm. The entire left ovary and the majority of the right ovary were replaced with tumors. A left oophorectomy was then performed, which showed a diffuse infiltrative round blue cell tumor with the sparing of primordial ovarian follicles. Tumor cells were positive for CD45, CD20, and PAX5 and showed a high Ki-67 proliferation index, consistent with DLBCL. The tumor cells were also positive for CD10 and B-cell lymphoma antigen 6 (BCL6), consistent with the germinal center subtype. Additional stains performed to rule out other subtypes of DLBCL were all negative including CD30, c-MYC, human herpesvirus-8 (HHV-8), anaplastic lymphoma kinase 1 (ALK1), and Epstein-Barr virus expressing mRNA (EBER). CD138, a plasma cell marker, was positive in the tumor cells. *MYC* gene rearrangement and amplification tests were negative. Fluorescence in situ hybridization studies were suggestive of the loss of the *MYC* gene region or the loss of chromosome 8. Bilateral bone marrow and cerebrospinal fluid samples were negative for the disease. A positron emission tomography/computed tomography scan confirmed Murphy stage III with nodal involvement above and below the diaphragm plus extranodal involvement.

The patient was started on cyclophosphamide, vincristine, and prednisone (COP) reduction with an overall reduction of 62% of tumoral masses. The keloid-like lesion in the neck had a near-complete resolution by day 8 of treatment (Figure 1B). Even though she was considered to have standard-risk B-cell malignancy, due to the extranodal involvement of the ovaries and the finding of CD138 positivity by immunohistochemistry (proposed to be an independent negative prognostic marker in cases of DLBCL) [6], rituximab was added to standard B-cell therapy.

## Discussion

Ovarian malignancies most commonly occur in females who have reached reproductive maturity. When ovarian malignancy occurs in children, they are more frequently non-epithelial cell tumors such as germ cell tumors and sex cord-stromal tumors [7]. They account for 1% of all malignancies in children from birth to 17 years [2].

Pediatric NHL comprises 8% of childhood malignancies with DLBCL comprising 10%-20% of NHL [1-3]. Most commonly, NHL will present in the mediastinum, neck, or abdomen [8]. Lymphomas of the ovary in the pediatric population are extremely rare occurrences. They can be primary or secondary, with the latter presenting in the setting of widely disseminated disease or as a presenting feature of extra-ovarian disease [9,10]. NHL primarily involving the ovary is even rarer with scant literature review available, limited to case reports.

Primary ovarian lymphoma is uncommon due to the lack of lymphoid tissue present in the ovaries. One theory of its origin includes lymphoma arising from lymphocytes in the vasculature near the hilum or corpus luteum. Another theory is that they could be the result of reactive lymphocytosis secondary to various gynecologic pathologies [11].

Paraneoplastic syndromes are a vast array of disorders that occur in the presence of a malignancy but are not directly associated with the primary tumor or metastasis. They can often be one of the first signs of an underlying malignancy as was the case with the patient presented in this report. Similarly, NHL has been associated with a wide spectrum of paraneoplastic syndromes due to cytokine responses, autoimmune activity, or ectopic hormone production [12]. In this case, the patient presented with a dermatologic lesion consisting of a fibrosing T-cell predominant inflammatory infiltrate that restricted the range of motion of the neck. A few of the dermatologic paraneoplasms that have been associated with NHLs include Sweet’s syndrome, pemphigus, eosinophilic fasciitis, granuloma annulare, ichthyosis, and other non-specific inflammatory conditions of the skin [12,13]. We considered the possibility of a paraneoplastic case of scleroderma given the clinical appearance, presence of dysphagia (hinting at esophageal peristalsis), and histopathological findings. Rongioletti and Rebora reported a case of scleromyxedema or generalized lichen myxedematosus, which was characterized by an extensive symmetrical eruption of small, waxy papules that progress to sclerosis and the induration of the skin, usually on the face, neck, upper trunk, arms, and thighs [14]. They proposed four features to define the diagnosis: (1) generalized papular and sclerodermoid eruption; (2) mucin deposition, fibroblast proliferation, and fibrosis; (3) monoclonal gammopathy; and (4) the absence of thyroid disease. When a case showed the typical clinical and histopathological features without monoclonal gammopathy, they called it an atypical form of scleromyxedema. Our patient met only the criteria of generalized sclerodermoid eruption, and the histopathological findings were suggestive of early scleroderma. Should the POL have gone undiagnosed and untreated, the lesion may have evolved to have a more definitive histopathological characterization. The fact that the lesion and the induration around the neck improved upon the initiation of treatment confirms the relationship of a paraneoplastic syndrome.

## Conclusions

This case illustrates a unique and rare occurrence of bilateral DLBCL of the ovaries with associated paraneoplastic scleroderma-like skin induration as the leading presentation. The authors caution physicians to consider potential primary ovarian lymphomas or other cancers in the presence of unexplained skin abnormalities, especially sclerotic lesions.
